# Dissecting Generalizability and Actionability of Disease-Associated Genes From 20 Worldwide Ethnolinguistic Cultural Groups

**DOI:** 10.3389/fgene.2022.835713

**Published:** 2022-06-24

**Authors:** Emile R. Chimusa, Shatha Alosaimi, Christian D. Bope

**Affiliations:** ^1^ Division of Human Genetics, Department of Pathology, University of Cape Town, Medical School Cape Town, Cape Town, South Africa; ^2^ Institute of Infectious Disease and Molecular Medicine, University of Cape Town, Cape Town, South Africa; ^3^ Department of Mathematics and Computer Science, University of Kinshasa, Kinshasa, Congo; ^4^ Centre for Bioinformatics, Department of Informatics, University of Oslo, Oslo, Norway

**Keywords:** actionable gene, incidental finding, whole-genome sequencing, next-generation sequencing, genetic diversity, population genetics actionable gene, population genetics

## Abstract

Findings resulting from whole-genome sequencing (WGS) have markedly increased due to the massive evolvement of sequencing methods and have led to further investigations such as clinical actionability of genes, as documented by the American College of Medical Genetics and Genomics (ACMG). ACMG’s actionable genes (ACGs) may not necessarily be clinically actionable across all populations worldwide. It is critical to examine the actionability of these genes in different populations. Here, we have leveraged a combined WES from the African Genome Variation and 1000 Genomes Project to examine the generalizability of ACG and potential actionable genes from four diseases: high-burden malaria, TB, HIV/AIDS, and sickle cell disease. Our results suggest that ethnolinguistic cultural groups from Africa, particularly Bantu and Khoesan, have high genetic diversity, high proportion of derived alleles at low minor allele frequency (0.0–0.1), and the highest proportion of pathogenic variants within HIV, TB, malaria, and sickle cell diseases. In contrast, ethnolinguistic cultural groups from the non-Africa continent, including Latin American, Afro-related, and European-related groups, have a high proportion of pathogenic variants within ACG than most of the ethnolinguistic cultural groups from Africa. Overall, our results show high genetic diversity in the present actionable and known disease-associated genes of four African high-burden diseases, suggesting the limitation of transferability or generalizability of ACG. This supports the use of personalized medicine as beneficial to the worldwide population as well as actionable gene list recommendation to further foster equitable global healthcare. The results point out the bias in the knowledge about the frequency distribution of these phenotypes and genetic variants associated with some diseases, especially in African and African ancestry populations.

## Introduction

NGS analysis contributed to the improvement of patient treatment and clinical care. This development has bridged the gap between healthcare and genomics. Furthermore, variant calling is an important aspect of genomics studies as polymorphism information can be used to influence the discovery of actionable pathogenic variants and therefore impact important clinical decisions. Currently, the definition of actionable pathogenic variants varies among scholars (Bope et al., 2019).

The Clinical Genome Resource (ClinGen) presents actionability as clinically prescribed interventions to a genetic disorder that is effective for prevention, lowered clinical burden or delay for a clinical disease, or improved clinical treatments and outcomes in a previously undiagnosed adult (Hunter et al., 2016). On the other hand, the 100,000 Genomes Project protocol presents actionable genes as variants that can significantly prevent (or result in illness or disability that is clinically significant, severely life-threatening, and clinically actionable) disease morbidity and mortality, if identified before symptoms become apparent. However, in any case, the classification of variants to be clinically actionable or not dependent and can only emerge during the process of seeking ethical approval for the study (Hunter et al., 2016).

Overall, in the current literature and most annotation databases, the classification of pathogenicity differs (Sherry et al., 2001; Wang et al., 2010; Landrum et al., 2016; McLaren et al., 2016). Dorschner et al. (1016) leveraged exome data of European and African populations to dissect actionable pathogenic variants, and the result shows that actionable pathogenic variants were disproportionate between European and African populations with an estimated frequency of approximately 3.4 and 1.2%, respectively. This indicates a deficit in the identification or categorization of pathogenic variants in African populations. A similar study conducted by Amendola et al. (2015) also confirmed the findings of Dorschner et al. (1016). One approach to define actionability is to combine many annotation pipelines during filtering and prioritization of mutations, in which casting vote can be applied respectively to allow better prediction of the targeted variant (Lebeko et al., 2017; Bope et al., 2019). Furthermore, on top of ethical approval, the ancestral/derived minor allele frequency of the variants, segregation evidence, and the number of patients affected with the variants and their status as a *de novo* mutation can highly be considered.

In this study, we provide a broad assessment of the possible actionability of variants known to be associated with the top four burden African diseases and a list of actionable genes from the American College of Medical Genetics and Genomics (ACMG) using WGS data of 20 worldwide ethnolinguistic cultural groups. This work aims to 1) perform variant join calling on publicly available data from the African Genome Variation and the 1000 Genomes Project to examine the evolutionary variation of pathogenic mutation; 2) perform disease-gene population structure; and 3) examine the heterozygosity ratio, the proportion of ancestral/derived alleles, and the distribution of minor allele frequencies based on selected known disease-genes from four predominant African burden diseases, HIV/AIDS, malaria, TB, and sickle cell disease, and a set of known actionable genes across 20 worldwide ethnolinguistic cultural groups. These diseases have uniquely shaped ethnolinguistic culturally specific groups and continental-specific genomic variations, and therefore offer unprecedented opportunities to map disease genes.

Our results in support with previous findings indicate higher genetic diversity in ethnolinguistic cultural groups from Africa, based on four African burden diseases and associated actionable genes. The results suggest the limitation of transferability or generalizability and support the use of personalized medicine as beneficial to each worldwide population or ethnolinguistic cultural group. In addition, our results point out the bias in the knowledge about the frequency distribution of these phenotypes and genetic variants associated with some diseases, especially in African and African ancestry populations, suggesting further examination of actionable gene lists to improve equitable global healthcare.

## Results

Based on the initial sample description of populations and country labels and leveraging the population culture and ethnolinguistic information (Gudykunst and Schmidt, 1987; Michalopoulos, 2012), we grouped 4,932 samples from their country labels into 20 independent ethnolinguistic cultural groups (Supplementary Table S1) and performed an independent joint call (see Materials and Methods), resulting in 90, 641, and 235 curated polymorphisms. We leveraged the dbSNPS database in extracting SNPs associated with 77, 50, 75, 460, and 114 genes known to be associated with tuberculosis, malaria, sickle cell disease, HIV, and ACMG’s actionable genes, respectively (Table 1), to examine the generalizability and actionability of these disease-associated genes from 20 worldwide ethnolinguistic cultural groups.

**TABLE 1:** Number of SNPs after quality control (QC) in each group of genes associated with HIV, TB, SCD, malaria, and actionable genes.

SNP	Gene	Disease
649,078	114	ACG
2,735,797	460	HIV
265,427	50	Malaria
4,455,648	75	SCD
2,513,341	77	TB

### Disease and Actionable Gene-Specific Population Structure

**FIGURE 1:**
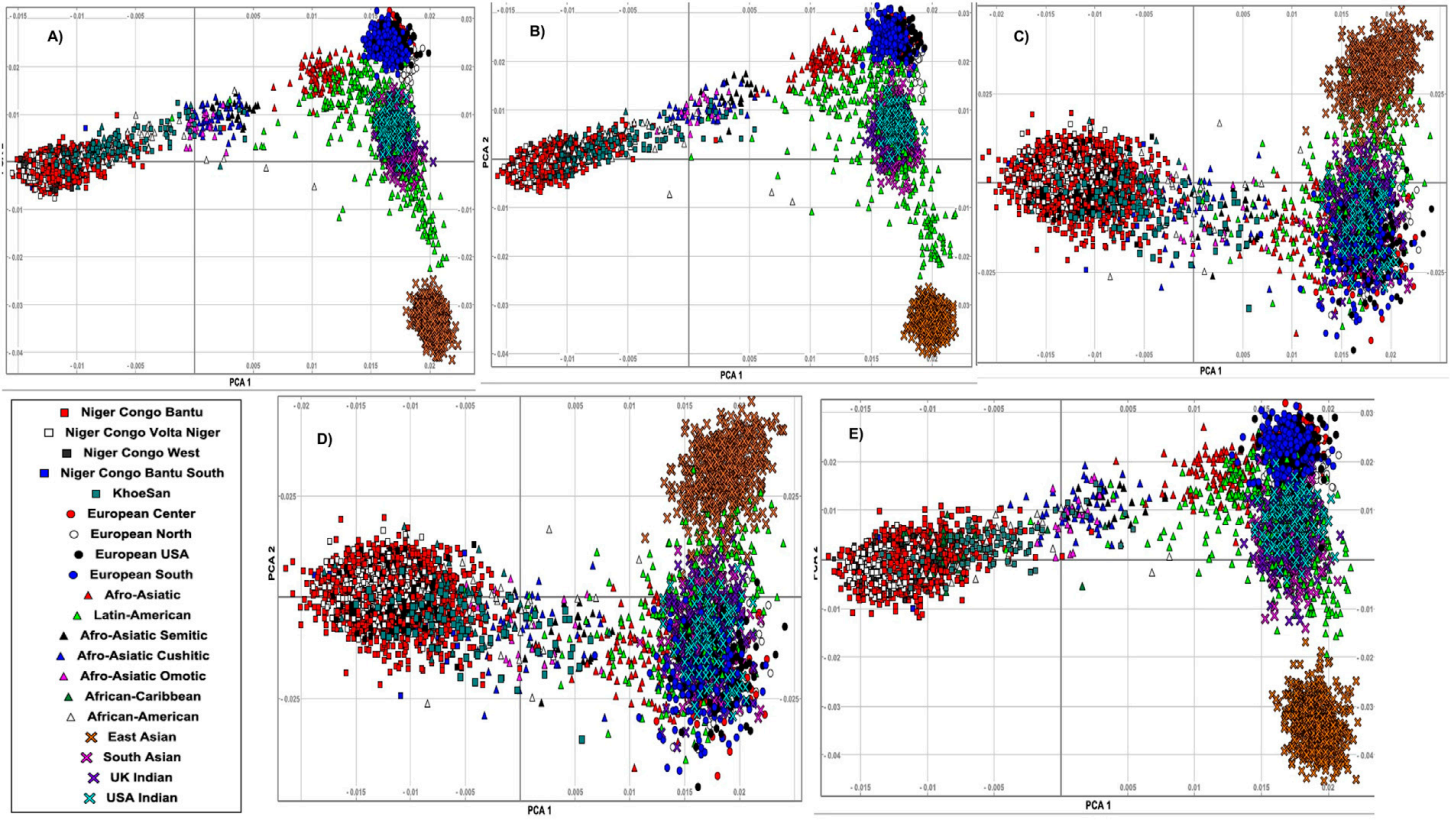
Principal component analysis (PCA) of genes associated with **(A)** HIV-specific, **(B)** TB-specific, **(C)** malaria-specific, **(D)** sickle cell disease-specific, and **(E)** ACG-specific SNPs and plots of the first and second eigenvectors for 20 ethnolinguistic cultural groups.

To better characterize the genetic relatedness, we first conducted principal component analysis (PCA) on whole-genome SNPs across all these 20 ethnolinguistic cultural groups (Supplementary Figure S1). Regardless of ethnolinguistic cultural groups, the results in Supplementary Figure S1 show a clear separation between African, European, Indian, and Eastern Asian groups. Second, based on the extracted disease-specific SNPs of different diseases, among these 20 different worldwide ethnolinguistic cultural groups (Materials and Methods), we performed principal component analysis (PCA). This PCA produces a set of orthogonal axes for which the remaining variances in the data are maximized by each successive dimension. Supplementary Table S2 illustrates the genetics distance (Fst) based on disease-specific variants among these 20 ethnolinguistic cultural groups. We present our gene-specific population structure results for HIV (Figure 1A), TB (Figure 1B), malaria (Figure 1C), sickle cell anemia (Figure 1D), and ACG (Figure 1E). Our results show that HIV variation is observed among Bantu, African–American, Khoesan, and Afro-related ethnolinguistic cultural groups, while the European group is clustering together (Figure 1A). Most ethnolinguistic cultural groups from Africa have the highest HIV gene-specific frequency (Figure 1A), confirming that HIV infection has a high incidence or prevalence among ethnolinguistic cultural groups from Africa compared to other ethnolinguistic cultural groups. Moreover, a variation in HIV-specific genes shows little overlap between/within ethnolinguistic cultural groups. The first principal component (PC) separates the European-related ethnolinguistic cultural group cluster and the African-related ethnolinguistic cultural cluster from one end to the other with the Afro-Asiatic ethnolinguistic cultural groups, the African–American, and one part of the Latin-Americans in the middle. The second principal component separates the European-related ethnolinguistic cultural cluster and the East Asian ethnolinguistic cultural group from one end to the other with the United Kingdom/United States–Indian group, the South Asian, and one part of the Latin-American ethnolinguistic cultural group in the middle. We also observe a cline between each axis. The dispersion of samples of HIV-specific genes along the lines suggests the existence of an admixture which may have occurred between ethnolinguistic cultural groups located on the same line and added to a strong local adaptation of HIV-specific genes among ethnolinguistic cultural groups located in the middle of each cline. One interesting observation is the intersection of the Latin-American ethnolinguistic cultural group with the Afro-Asiatic ethnolinguistic cultural groups on one side and the United Kingdom/United States–Indian and South Asian ethnolinguistic cultural groups on the other side which may indicate either a possible existence of HIV-specific actionable genes overlapping between these mentioned populations or a differing effect of these genes across these ethnolinguistic cultural groups. As for HIV, a variation in TB-specific genes was observed among Bantu and Khoesan and Afro-related ethnolinguistic cultural groups (Figure 1B), while European groups are clustering together, except in North European (explaining the known high incidence of TB in Central and North Europe). As the same observation for TB is similar to HIV, then the same comment applies for TB as well. Malaria-specific worldwide ethnolinguistic cultural groups’ genetic structure (Figure 1C) shows that ethnolinguistic cultural groups from Africa and African–American ethnolinguistic cultures are still separated from the rest of the other ethnolinguistic cultural groups. United Kingdom/United States–Indians and Afro-related, Latin-American, and all Europeans are clustering together based on malaria-specific genes, low prevalence, and/or absence of malaria in their geographic regions, indicating that the malaria-specific genes found in one of these aforementioned populations may not be found in the other population. East/South Asians are clustering apart from ethnolinguistic cultural clusters from Africa and Europe continents. While it is known that malaria has a high prevalence among African and Asian populations, the separate cluster between them may indicate different patterns of linkage disequilibrium, geographic location, and genetic variation in malaria-specific genes. As expected, since malaria and sickle cell disease are known to be genetically correlated, similar results for Malaria are observed with sickle-cell disease-specific genes (Figure 1D). The population structure on ACG-specific genes reveals that Africa and European-related ethnolinguistic cultural groups, East-Asian ethnolinguistic cultural groups, and United Kingdom/United States–Indian and South Asian ethnolinguistic cultural groups are separated and clustered in three different clusters (Figure 1E). We observed that African–American and Afro-related ethnolinguistic cultural groups are in the convex of these three clusters (Figure 1E), justifying that they are the result of the admixture of these ethnolinguistic cultural groups considered geographic ancestral populations. In addition, Latin-America is close to European and South Asian clusters, as seen from the results of the admixture, and they are mainly in the convex between East-Asian, South-Asian, and European groups, and a bit distant to the ethnolinguistic cultural groups from Africa. This result indicates that the transferability or generatability of the actionability of these ACG genes may have differing effects across 20 worldwide ethnolinguistic cultural groups.

### Proportion of Pathogenic Polymorphisms Within Disease-Associated Genes

**FIGURE 2:**
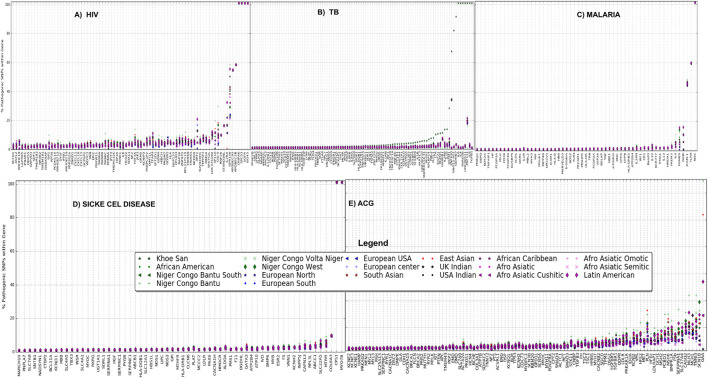
Proportion of pathogenic variants within **(A)** HIV-specific, **(B)** TB-specific, **(C)** malaria-specific, **(D)** sickle cell disease-specific, and **(E)** ACG-specific (actionable genes) genes among all 20 ethnolinguistic cultural groups.

Ethnolinguistic cultural groups from Africa including Bantu and Latin-American and Afro-related groups have a considerable high proportion of pathogenic variants in these HIV-specific genes (Figure 2A). We observe that the Khoesan ethnolinguistic cultural group has a high proportion of pathogenic variants within TB-specific genes (Figure 2B). Latin-American, Afro-Asiatic, and African ancestry (African diaspora)-related ethnolinguistic cultural groups have a high proportion of pathogenic variants (Figure 2B). The low proportion of pathogenic variants is observed across all malaria-specific genes in Bantu, Afro-Asiatic, and Latin-American ethnolinguistic cultural groups (Figure 2C); however, except for toll-like receptor 9 (*TLR9*), *FREM3*, *IL4*, *ICAM-1*, and nitric oxide synthase 1 (neuronal), the Bantu-related ethnolinguistic cultural groups and Latin-Americans have a high proportion of pathogenic variants (Figure 2C). Bantu, Afro-related ethnolinguistic cultural groups, and Latin America have a similar low proportion of pathogenic variants in most of the sickle cell disease-specific genes, except in *MY O 7B*, *CPS1*, *COL6A3*, *MTRR*, *SLC22A5*, *ABCC1*, and *RPL3L* (Figure 2D). We observed a considerable high proportion of pathogenic variants within ACG-specific genes from ethnolinguistic cultural groups out of the African continent including Latin America, Afro-Asiatic, and European-related ethnolinguistic cultural groups (Figure 2E), while few genes show a high proportion of pathogenic variants in Niger-Bantu and African–American groups (Figure 2E).

### Distribution of Gene-Specificity in SNP Frequencies

**FIGURE 3:**
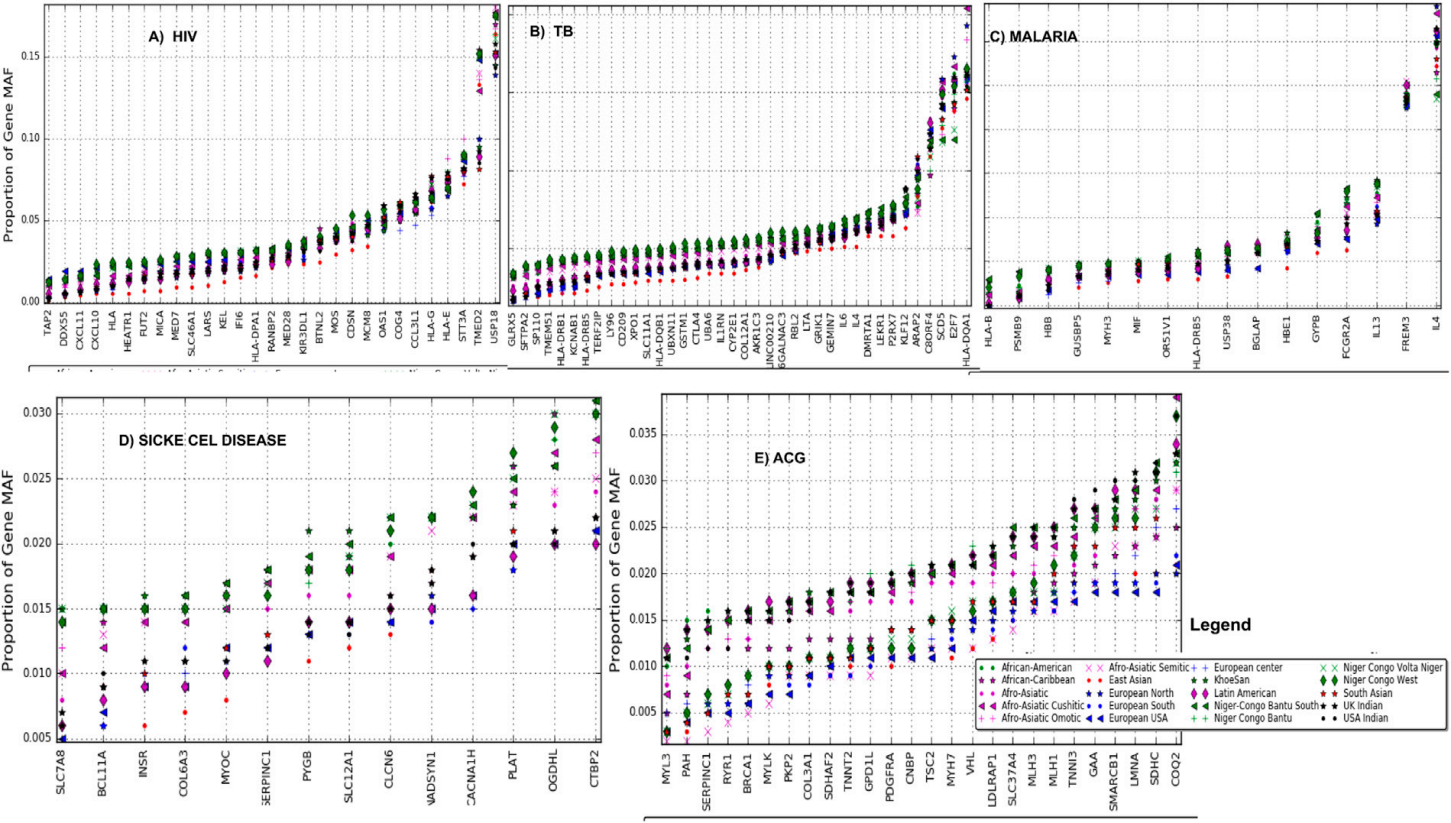
Gene-specificity in SNP minor allele frequency: the distribution of the minor allele frequency at the gene level **(A)** HIV, **(B)** TB, **(C)** malaria, **(D)** sickle cell disease, and **(E)** ACG (actionable genes) among all ethnolinguistic cultural groups.

We observed variations in the distribution of MAF at rare variants within MAF bin 0.0–0.05 among these 20 ethnolinguistic cultural groups in four African burden diseases (Supplementary Figures S2A–D) and ACMG’s actionable genes (Supplementary Figure S2E). *BTNL2*, *MOS*, *CDSN*, *USP18*, *MCM8*, *OAS1*, *COG4*, *CCL3L1*, *HLA-G*, *HLA-E*, *STT3A*, *TMED2*, and *USP18* have HIV gene-specificity in SNP frequencies ranging between 5 and 15% (Figure 3A) and those ethnolinguistic cultural groups from Africa have the highest. A total of 33 genes have TB gene-specificity in SNP frequencies between 5 and 20% of which all ethnolinguistic cultural groups from Africa have the highest (Figure 3B), suggesting that these genes may harbor common effects and contributions to TB among African ethnolinguistic cultural groups. The distribution of malaria gene-specificity in SNP frequencies from Figure 3C suggests that four genes include *GYPB*, *FCGR2A*, *IL13*, and *FREM3* with gene-specificity ranging between 4 and 15%, while all sickle cell disease-related genes (Figure 3D) show low gene-specificity in SNP frequencies ranging between 0.1 and 0.3% among all 20 ethnolinguistic cultural groups, but all ethnolinguistic cultural groups from Africa have the highest frequencies. The distribution of ACG-gene-specificity in SNP frequencies in Figure 3E indicates that all ACG genes have gene-specificity in SNP frequencies lower than 0.4% in all 20 ethnolinguistic cultural groups. However, the gene-specificity in SNP frequencies from most of the ethnolinguistic cultural groups from Africa are higher than those from non-African ethnolinguistic cultural groups, supporting a potential difference effect and contribution of these actionable genes among worldwide ethnolinguistic cultural groups. Supplementary Table S3 shows the details of gene-specificity in SNP frequencies of these ACG and disease burdens across all these 20 ethnolinguistic cultural groups.

### Gene-Specific in Proportion of Derived Alleles and Relationship Between Derived and Ethnolinguistic Cultural-Specific Minor Allele Frequency

Derived alleles are more often minor alleles (<50% allele frequency) and associated with risk than ancestral alleles (32). As for the variation observed in the distribution of MAF at rare variants at low ethnolinguistic and cultural-specific minor allele frequencies (ranging between 0.0 and 0.1, Supplementary Figure S3), high variation in the proportion of derived alleles can be observed in HIV (Supplementary Figure S3A), TB (Supplementary Figure S3B), malaria (Supplementary Figure S3C), and sickle cell disease (Supplementary Figure S3D), and a set of actionable genes (Supplementary Figure S3E) across all ethnolinguistic cultural groups from Africa compared to the rest of the other ethnolinguistic cultural groups, and that most of the ethnolinguistic cultural groups from Africa have the highest proportion of derived alleles in the range of minor allele frequency bin (0.0–0.1) (Supplementary Figure S3A), indicating that different mutations and possible selections occurred in rare variants within genes associated with these four African burden diseases, and ACMG’s actionable genes play critical roles and that ethnolinguistic and cultural-specific risk alleles may differentially contribute to the phenotypic variations and clinical outcomes.

**FIGURE 4:**
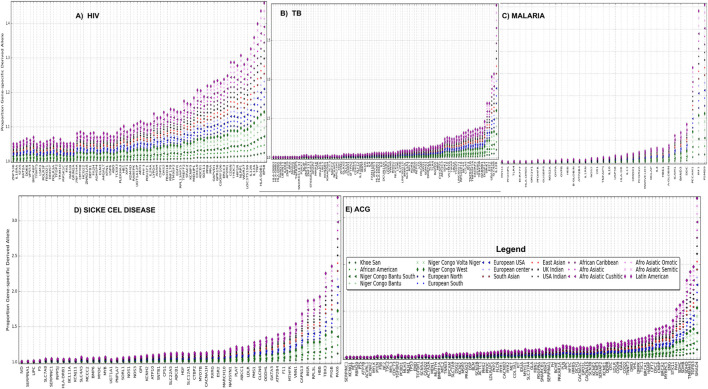
**(A)** HIV, **(B)** TB, **(C)** malaria, **(D)** sickle cell disease, and **(E)** ACG gene-specific proportion of derived alleles across 20 worldwide ethnolinguistic cultural groups.

To obtain gene-specific proportions of derived alleles, derived allele frequencies were aggregated for all SNPs associated with each of these disease-specific genes (see Materials and Methods). For all African burden diseases including HIV (Figure 4A), TB (Figure 4B), malaria (Figure 4C), and sickle cell diseases (Figure 4D), we observe that Latin America and most of Afro-Asiatic, Bantu, and Khoesan ethnolinguistic cultural groups have a considerable and consistently high proportion of gene-specific derived alleles. We observe a consistent high ACG-gene-specific allele in Latin America and most Afro-related ethnolinguistic cultural groups following most of European-related ethnolinguistic cultural groups (Figure 4E), while a low ACG-gene-specific allele is observed in most of African ethnolinguistic cultural groups. One can expect actionable genes to have a high proportion of derived alleles; however, this is not the case for most of African ethnolinguistic cultural groups, indicating that the current ACG genes were primarily tailored for non-African ethnolinguistic cultural groups. A full list of the ethnolinguistic and cultural gene-specific proportions of derived alleles based on genes associated with these four African burden diseases and ACMG’s actionable genes can be found in Supplementary Table S4.

### Genetic Diversity: Observed and Expected Heterozygosity

**FIGURE 5:**
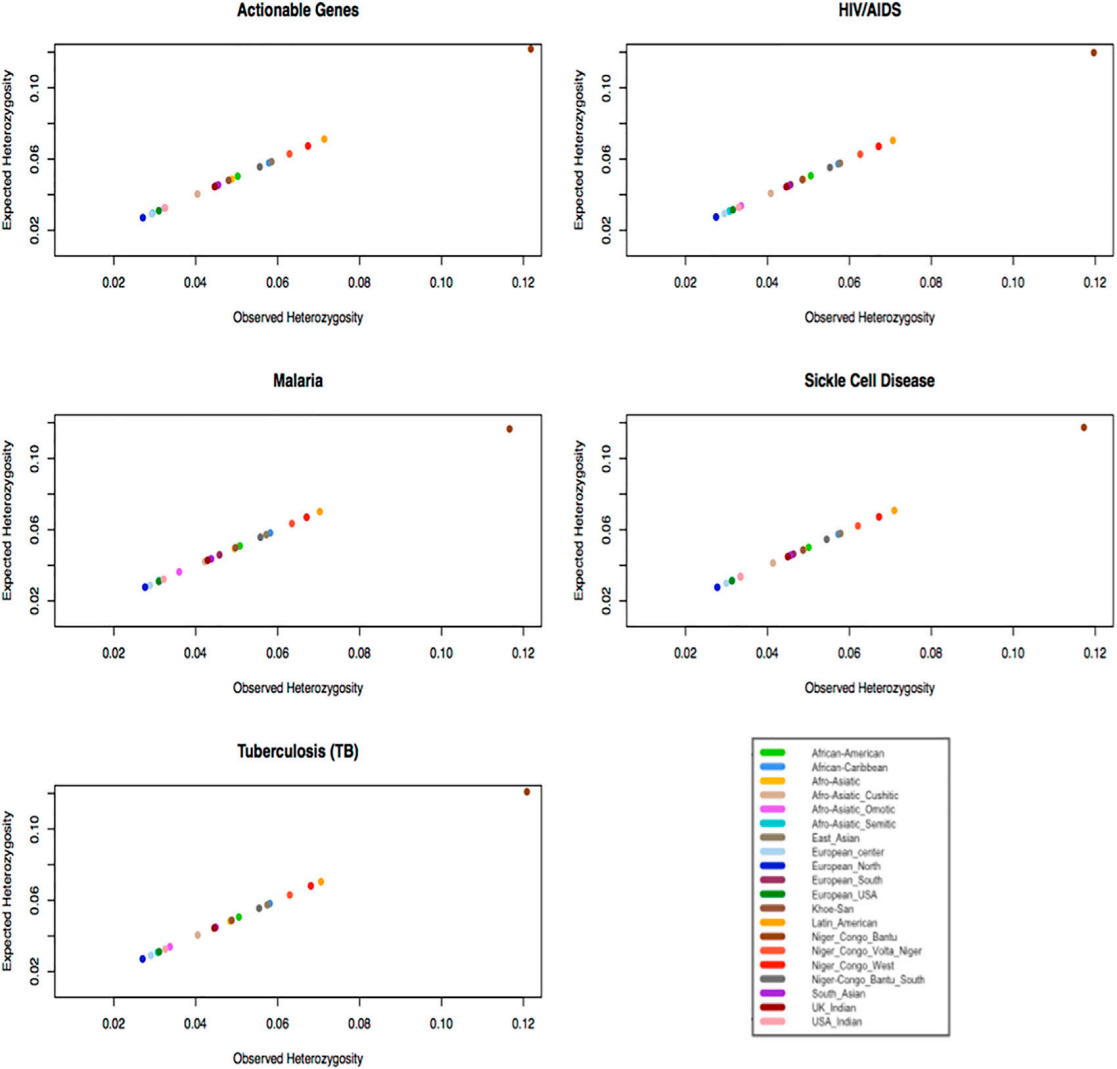
Plot expected heterozygosity as a function of observed heterozygosity per gene of specific diseases within ethnolinguistic cultural groups.

Gene diversity consists of two elements including the abundance (or evenness) of the alleles and the number of alleles. The abundance (or evenness) of the alleles and the number of alleles would increase the expected heterozygosity. If an ethnolinguistic cultural group consists of an excess of homozygotes for different alleles, this leads to low-observed heterozygosity. In Figure 5, we observe that ethnolinguistic cultural groups from Africa, particularly Bantus and Khoesan, have the highest gene diversity in HIV, TB, malaria, sickle cell disease, and ACG-associated variants (Supplementary Table S5). This result supports the highest genetic diversity found in individuals and communities across the African continent and that the use of personalized medicine will be beneficial to both the continent and world.

## Discussion

In this study, we conducted a joint call of 4,932 samples representing 20 worldwide ethnolinguistic cultural groups (Supplementary Table S1), to examine the generalizability and actionability of 77, 50, 75, 460, and 114 genes known to be associated with tuberculosis, malaria, sickle cell disease, HIV, and ACG, respectively (Table 1). To examine the generalizability and actionability of genes, we investigated the distribution of (Bope et al., 2019) gene-specificity in SNP frequencies, (Hunter et al., 2016), gene-specificity in the proportion of derived alleles, and (Sherry et al., 2001) gene-specificity in pathogenic mutations. In addition, population-specific genetic structures and expected heterozygosity were observed in all associated SNPs within genes.

The results of HIV/TB indicated that ethnolinguistic cultural groups including Bantu, Latin American, and Afro-Asiatic have the highest proportion of pathogenic variants based on 483 HIV-specific genes. From 77 TB-specific genes, we observed that Latin American and Afro-Asiatic ethnolinguistic cultural groups have the highest proportion of pathogenic variants, important among all African and African diaspora ethnolinguistic cultural groups, and only Khoesan has a high proportion of pathogenic variants within TB-specific genes. Most ethnolinguistic cultural groups from Africa (Bantu and Khoesan) have the highest HIV and TB gene-specific frequency, indicating that HIV disease risk is prevalent among African ethnolinguistic cultural groups compared with other ethnolinguistic cultural groups. Our result identifies *BTNL2*, *MOS*, *CDSN*, *USP18*, *MCM8*, *OAS1*, *COG4*, *CCL3L1*, *HLA-G*, *HLA-E*, *STT3A*, *TMED2*, and *USP18* to have HIV gene-specificity in SNP frequencies ranging between 5 and 15% and those ethnolinguistic cultural groups from Africa have the highest. In addition, 33 genes have TB gene-specificity in SNP frequencies ranging between 5 and 20% of which all African ethnolinguistic cultural groups have the highest frequencies. This suggests that these genes may harbor a common effect and contribution to TB/HIV among African ethnolinguistic cultural groups. Furthermore, HIV/TB gene-specificity has a high proportion of derived alleles at low minor allele frequency (0.0–0.1) from African ethnolinguistic cultural groups and that these proportions of derived alleles vary among African ethnolinguistic cultural groups, suggesting a possible challenge in enabling cross-population actionable gene transferability and possible implementation of precision medicine within different ethnolinguistic cultural groups from Africa.

The results of malaria and sickle cell disease indicate the absence of pathogenic variants in most of the European-related ethnolinguistic cultural groups and a low proportion of pathogenic variants across all malaria-specific genes in Bantu, Afro-Asiatic, and Latin American ethnolinguistic cultural groups, except for toll-like receptor 9 (*TLR9*), *FREM3*, *IL4*, *ICAM-1*, and nitric oxide synthase 1 (neuronal), indicates that Bantu and Latin America ethnolinguistic cultural groups have a high proportion of pathogenic variants. Furthermore, Bantu, Afro-Asiatic, and Latin American ethnolinguistic cultural groups have a similar low proportion of pathogenic variants in most sickle cell disease-specific genes, except in *MY O 7B*, *CPS1*, *COL6A3*, *MTRR*, *SLC22A5*, *ABCC1*, and *RPL3L*. We identify four genes including *GYPB, FCGR2A, IL13,* and *FREM3* with malaria gene-specificity in SNP frequencies ranging between 4 and 15%, while all sickle cell disease-related genes have low gene-specificity in SNP frequencies ranging between 0.1 and 0.3% among all 20 ethnolinguistic cultural groups, but all African and diaspora ethnolinguistic cultural groups have the highest in that range.

The result on ACG showed a considerably high proportion of pathogenic variants within ACG-specific genes from non-African ethnolinguistic cultural groups including Latin American, Afro-Asiatic, and European compared to most of African-related ethnolinguistic cultural groups. This result justifies and indicates that the actionability of these ACG genes may have heterogeneous effects on worldwide ethnolinguistic cultural groups, unraveling cross-ethnic group transferability and generalizability to diverse ethnic groups, particularly African from ACG-specific actionable genes daunting. Our result indicates that all ACG genes have gene-specificity in SNP frequencies lower than 0.4% in all 20 ethnolinguistic cultural groups. However, the gene-specificity in SNP frequencies from most of African ethnolinguistic cultural groups are higher than those from non-African ethnolinguistic cultural groups, supporting the potential common effect and contribution of these actionable genes to non-African ethnolinguistic cultural groups. A high ACG-gene-specific derived allele was observed in Latin-American and most Afro-related ethnolinguistic cultural groups following most of European-related ethnolinguistic cultural groups, while a low ACG--specific derived allele is observed in most of African ethnolinguistic cultural groups.

We leveraged the dbSNP database to extract SNPs associated with these genes per disease. The obtained SNPs per disease were thus extracted from the whole phased data containing 4,932 samples of these 20 ethnolinguistic cultural groups, yielding five disease-specific phased haplotype datasets. From these phased haplotype data, we conducted disease gene-specific population structure, and we examined the distribution and relationship of derived and minor allele frequency and estimated the expected and observed heterozygosity.

The result of this study suggests significant genetic variations among all non-European ethnolinguistic cultural groups, mostly African ethnolinguistic cultural groups, while all European ethnolinguistic cultural groups are genetically and consistently clustering together based on these diseases or actionable-specific variants, suggesting limitations of cross-population transferability of actionable or medically relevant genes, given the exceptional polygenicity of human traits. Furthermore, the result indicates that African and African diaspora ethnolinguistic cultural groups, particularly Bantus and Khoesan ethnolinguistic cultural groups, have the highest gene diversity in HIV, TB, malaria, sickle cell disease, and ACG-associated variants. This supports the highest genetic diversity found in individuals and communities across the African continent. Based on these findings, the use of personalized medicine including African genomics will be beneficial to both the continent and world. One of the limitations of this finding is that although these results depend greatly on laboratory experiments, the distribution of actionable genes across populations may depend on continuous genetic diversity, natural selection, and genetic drift. Such study paves the way for a continuous analysis of disease-specific actionable genes and their genetic mechanism underpinning those diseases.

## Concluding Remarks

In conclusion, our findings suggest the highest genetic diversity in African ethnolinguistic cultural groups in the four African burden diseases and ACMG’s actionable genes, and that the distribution of gene-specificity (Bope et al., 2019) in SNP frequencies (Hunter et al., 2016), in the proportion of derived alleles, and (Sherry et al., 2001) in pathogenic mutations based on the obtained 77, 50, 75, 460, and 114 genes was known to associate with tuberculosis, malaria, sickle cell disease, HIV, and ACMG’s actionable genes, respectively, indicating significant variation across 20 worldwide ethnolinguistic cultural groups. This suggests (Bope et al., 2019) the limitation of transferability or generalizability; however, the use of personalized medicine will be beneficial to both the African continent and worldwide (Hunter et al., 2016), enabling a recommendation for an African-specific actionable list of genes which will further improve African and diaspora healthcare.

## Materials and Methods

### Data Description and Quality Check

The data Binary Alignment Map (BAM) files were obtained from the 1000 Genomes Project (1KGP) (Siva, 2008) and the African Genome Variation Project (AGVP) (Gurdasani et al., 2015), which has recently characterized the admixture across 18 ethnolinguistic groups from sub-Saharan Africa as shown in Supplementary Table S1. A quality control check was conducted on the BAM files using SAMtools (Li et al., 2009). After quality check, a total of 2,504 BAM files from the 1000 Genomes Project and 2,428 BAM files from the AGVP were retained. Based on initial sample description population and country labels, we used the population culture and ethnolinguistic information (Gudykunst and Schmidt, 1987; Michalopoulos, 2012) to group populations from the country label into 20 ethnolinguistic cultural groups (Supplementary Table S1). Supplementary Figure S1 illustrates the genetics relatedness and variation of these 20 ethnolinguistic cultural groups, supporting previous findings (Siva, 2008; Chimusa et al., 2015; Gurdasani et al., 2015; Choudhury et al., 2020), and Supplementary File 1 illustrates the genetics distance (Fst) based on disease-specific variants among the 20 ethnolinguistic cultural groups.

### Variants Discovery Analysis and Annotation

LoFreq, a variant calling tool, was used to conduct joint calls across 4,932 samples in 20 worldwide ethnolinguistic cultural groups. The resulting variant sets of all 4,932 samples in the VCF file were filtered using SAMtools, and 4,932 samples remained and were considered for downstream analysis.

The resulting joint call VCF file of 4,932 samples and samples were split into 20 VCF files per ethnolinguistic cultural group as listed in Supplementary Table S1. The independent gene-based annotation for each VCF dataset to determine whether SNPs cause protein-coding change and produce a list of amino acids that are affected was conducted using ANNOVAR (Wang et al., 2010). The following setting was used in ANOVA: the population frequency and pathogenicity for each variant were obtained from 1000 Genomes exome, Exome Aggregation Consortium (ExAC), targeted exon datasets, and COSMIC. Gene functions were obtained from RefGene, and different functional predictions were obtained from ANNOVAR’s library, which contains up to 21 different functional scores including SIFT (Ng et al., 2006), LRT (Schwarz et al., 2010), MutationTaster (Reva et al., 2011), MutationAssessor (Shihab et al., 2013), FATHMM and FATHMM-MKL (Liu et al., 2011), RadialSVM (Choi and Chan, 2015), LR (Kim et al., 2017), PROVEAN (Kim et al., 2017), MetaSVM (Dong et al., 2015), MetaLR (Rentzsch et al., 2018), CADD (Davydov et al., 2010), GERP++ (Quang et al., 2014), DANN (Jagadeesh et al., 2016), M-CAP (Ionita-Laza et al., 2016), Eigen (Lu et al., 2015), GenoCanyon (Adzhubei et al., 2010), Polyphen2-HVAR and HDIV (Doerks et al., 2002), PhyloP (Garber et al., 2009), and SiPhy (Loh et al., 2016a). In addition, conservative and segmental duplication sites were included, and the dbSNP code and clinical relevance were reported in dbSNP. From each resulting functional annotated dataset, we independently filtered for the predicted functional status, of which each predicted functional status is of “deleterious” (D), “probably damaging” (D), “disease-causing-automatic” (A), or “disease-causing” (D). The selection of mutations was carried out using the following approach: first, the casting vote approach was implemented in our custom Python script, to retain only a variant if it had at least 17 predicted functional status “D” or “A” out of 21 was used and second, the retained variants from each dataset were further filtered for rarity, exonic variants, and nonsynonymous mutations and with a high-quality call as described previously, yielding a final candidate list of predicted mutant variants in each subject group, including the replication group. We report on the aggregated SiPhy score from all identified mutant SNPs within the gene. The following sections provide details on how SNPs were mapped to genes.

### Phased and Haplotypes Inference

To increase the accuracy, the resulting VCF file, containing 4,932 samples of 20 ethnolinguistic cultural groups, was used to further conduct quality control in removing all structured, indel, multi-allelic variants and those with a low minor allele frequency (MAF <0.05) prior to phasing. We first phased and inferred the haplotypes using Eagle (Loh et al., 2016b) from the resulting curated data. We further compared site discordances between these haplotype panels and independently with their original VCF file before phasing. The only site with phase switch-errors showed discrepancies in MAF and was removed.

### Disease- and Actionable Gene-Specific Population Structure

We obtained the list of genes, known as medically actionable, and Actionable Genome Consortium (ACG) from https://www.coriell.org/1/NIGMS/Collections/ACMG-73-Genes. The list of genes associated with four major African diseases including malaria, TB, HIV, and sickle cell disease was collected from the GWAS Catalog (https://www.ebi.ac.uk/gwas/), and the extraction was based on phenotype classification and from databases such DisGeNET http://www.disgenet.org/and literature. We obtained 50, 77, 460, 75, and 114 genes known to be associated with tuberculosis, malaria, sickle cell anemia, HIV, and ACG, respectively. We leveraged the dbSNP database to extract SNPs associated with these genes per disease, as shown in Table 1. The obtained SNPs per disease were extracted from the whole phased data containing 4,932 samples of these 20 ethnolinguistic cultural groups, yielding five disease-specific phased haplotype datasets (Table 1).

To evaluate the extent of substructures within disease-specific polymorphism across worldwide ethnolinguistic cultural groups, we leverage each constructed disease-specific phased haplotype dataset, to perform genetic structure analysis based on principal component analysis (PCA) using smartpca, part of the EIGENSOFT 3.0 package (Patterson and Price, 2006). Genesis software http://www.bioinf.wits.ac.za/software/genesis was used to plot PCA.

### Proportion of Ancestral/Derived Alleles Among Risk-Conferring Alleles

Each of these four disease-specific phased haplotype datasets was used to analyze the fraction of derived and ancestral alleles and at-risk alleles within each ethnolinguistic cultural group. A previous work showed that derived alleles are more often minor alleles (<50% allele frequency) and associated with risk than ancestral alleles (Gorlova et al., 2012). Therefore, we define risk alleles as follows: if a gene is reported to increase the risk of disease (odd ratio >1) from either the DisGeNET or GWAS Catalog, the risk allele was defined as a minor allele (for all SNPs associated with the gene); otherwise (odd ratio <1), it is defined as a major allele (for all SNPs associated with the gene).

The SNP ancestral alleles were downloaded from the Ensembl, a 59 comparative 32 species alignment (Paten et al., 2008), and we further checked the SNPs for those present in the dbSNP database. Each of these four disease-specific phased haplotype datasets was further annotated using the VCFtools “fillOaa” script (Danecek et al., 2011) with the ancestral allele recorded using the “AA” INFO tag. For each disease-specific dataset, we determined the proportion of risk alleles that were ancestral or derived alleles. We first computed, for each SNP, the fraction of the ancestral allele, which was calculated by dividing the number of times the defined risk allele matched with the ancestral allele by the total number of copies of all alternative alleles across all samples (within each ethnolinguistic cultural group per disease) for a particular SNP. The fraction of the derived allele is equivalent to one minus the fraction of the ancestral allele. As mentioned earlier, derived alleles are more often minor alleles and associated with risk rather than ancestral alleles. Therefore, we investigated the relationship between the fraction of derived alleles, at-risk alleles, and ethnolinguistic cultural group SNP minor allele frequency. To this end, the alternative (minor) alleles were categorized into six bins, (0–0.05, >0.05–0.1, >0.1–0.2, >0.2–0.3, >0.3–0.4, and >0.4–0.5) with respect to each ethnolinguistic cultural dataset frequencies and independently computed the fractions of derived alleles in each bin. Furthermore, we computed the fraction of ancestral/derived alleles for all these known disease-specific genes. To this end, we aggregated the fraction of ancestral/derived alleles at the SNP-based level to gene, considering all SNPs located within the genes’ downstream or upstream region (Chimusa et al., 2015).

### Distribution of Minor Allele Frequency and Gene-Specificity in SNP Frequencies

To examine the extent of common variants across these 20 ethnolinguistic cultural groups within a specific disease (TB, HIV, sickle cell anemia, and malaria) and known actionable genes from ACG, the distribution of the minor allele frequency was investigated. To this end, the proportion of minor alleles was categorized into six bins (0–0.05, >0.05–0.1, >0.1–0.2, >0.2–0.3, >0.3–0.4, and >0.4–0.5) with respect to each ethnolinguistic cultural group with a disease. The minor allele frequency (MAF) per SNP for each category was computed using Plink software (Purcell et al., 2007). Furthermore, the fraction of gene-specific in SNP frequency for each gene was computed. To this end, the fraction of gene-specific SNP frequency was computed, assuming that SNPs in upstream and downstream within a gene region are close and possibly in linkage disequilibrium (LD). Minor allele frequency per SNP has aggregated a gene level.

### Aggregating SNP Summary Statistics at the Gene Level

SNP-specific allele frequencies or the proportion of ancestral/derived alleles from SNPs 40 kb downstream and upstream within a gene region as per the dbSNP database were aggregated (Chimusa et al., 2016). Under the null hypothesis, frequency/proportion $P_{\kappa }$ (k = 1,..., L) with a continuous distribution is uniformly distributed at the interval [0,1]. It follows that a parametric cumulative distribution function F can be chosen, and $P_{\kappa }$ can be transformed into quantile according to $q_{\kappa }=F^{-1}\left(P_{\kappa }\right)$ . The combined frequency/proportion $C^{P}=\;\frac{\sum_{\kappa }^{L}=1\;P_{\kappa }}{\sqrt{L}}$ is a sum of independent and identically distributed random variables $P_{\kappa }$ . To account for the independence assumption, given the correlation among neighboring genomic markers (Chimusa et al., 2016), we implement the Stouffer–Liptak method accounting for spatial correlations among SNPs within a gene or SNPs within a given sub-network. The overall statistic can be obtained by $P=\phi \left(C^{P}\right)$ , in which ϕ is the cumulative distribution function of the standard normal distribution.

### Key Points

• Personalized medicine including African genomics will be beneficial both to the continent and worldwide.
• Generalizability and transferability of actionable genes are challenging but will improve clinical population healthcare.
• Investigating the distribution of gene-specificity in SNP frequencies, gene-specificity in proportion of derived alleles, and gene-specificity in burden of pathogenic mutations will reveal population-specific actionable genes.

## Data Availability

The original contributions presented in the study are included in the article/Supplementary Material; further inquiries can be directed to the corresponding author.
