# Supplementary figures and images for: Dissecting Generalizability and Actionability of Disease-Associated Genes From 20 Worldwide Ethnolinguistic Cultural Groups
Source: Front Genet. 2022 Jun 24;13:835713. doi: 10.3389/fgene.2022.835713 (PMC9263835; doi:10.3389/fgene.2022.835713)

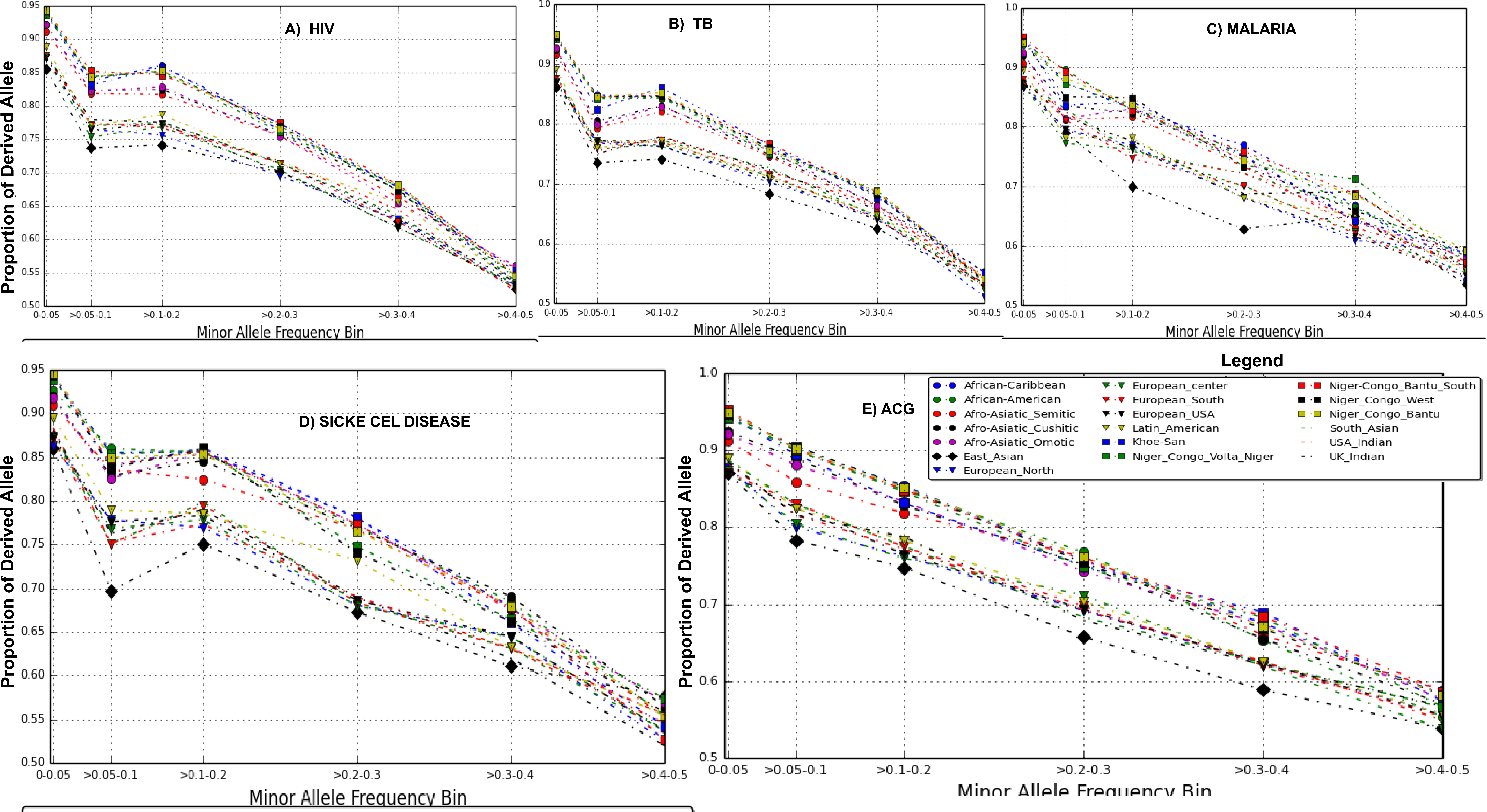

Supplement: Supplementary file 3 [file Image3.TIF]

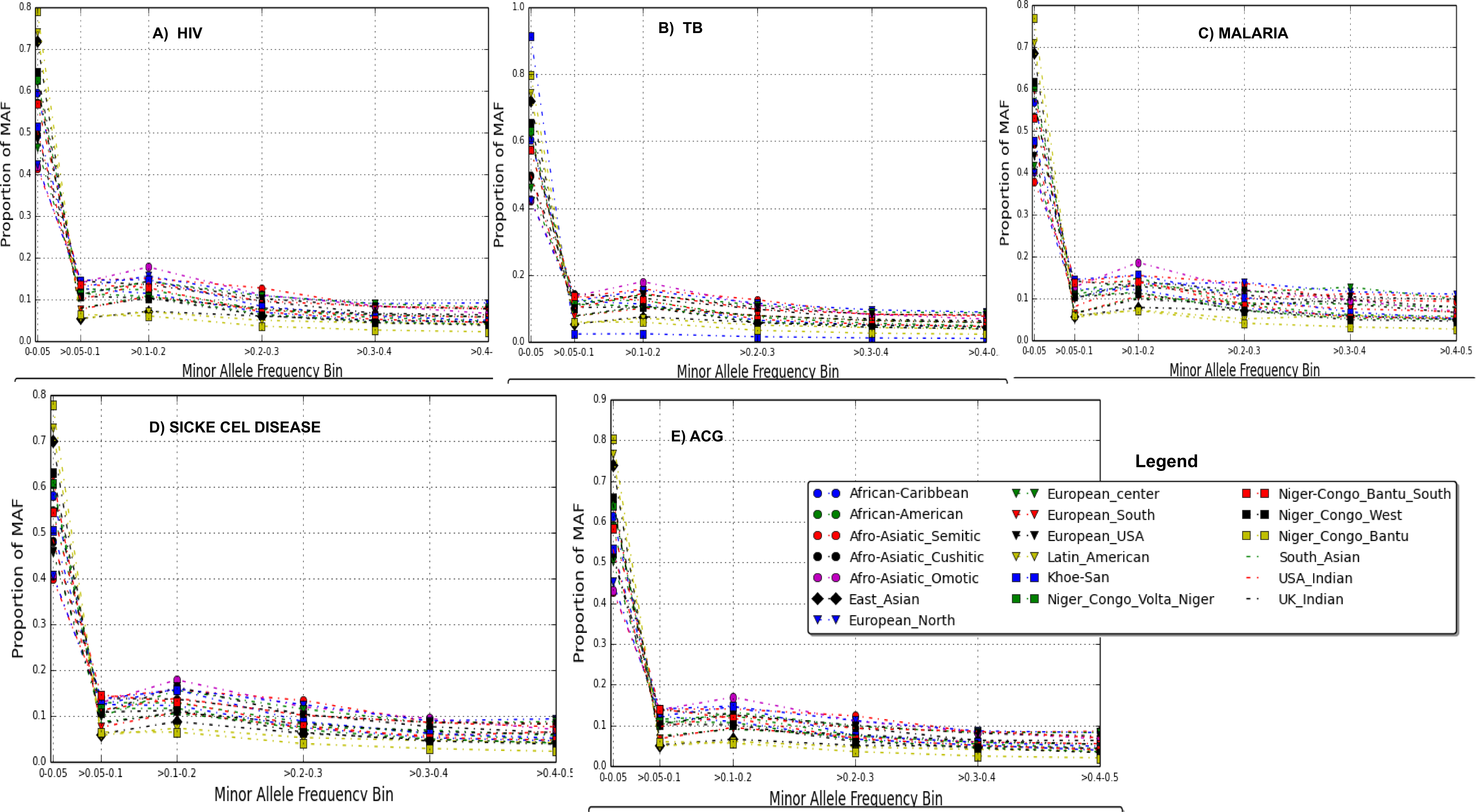

Supplement: Supplementary file 5 [file Image2.TIF]

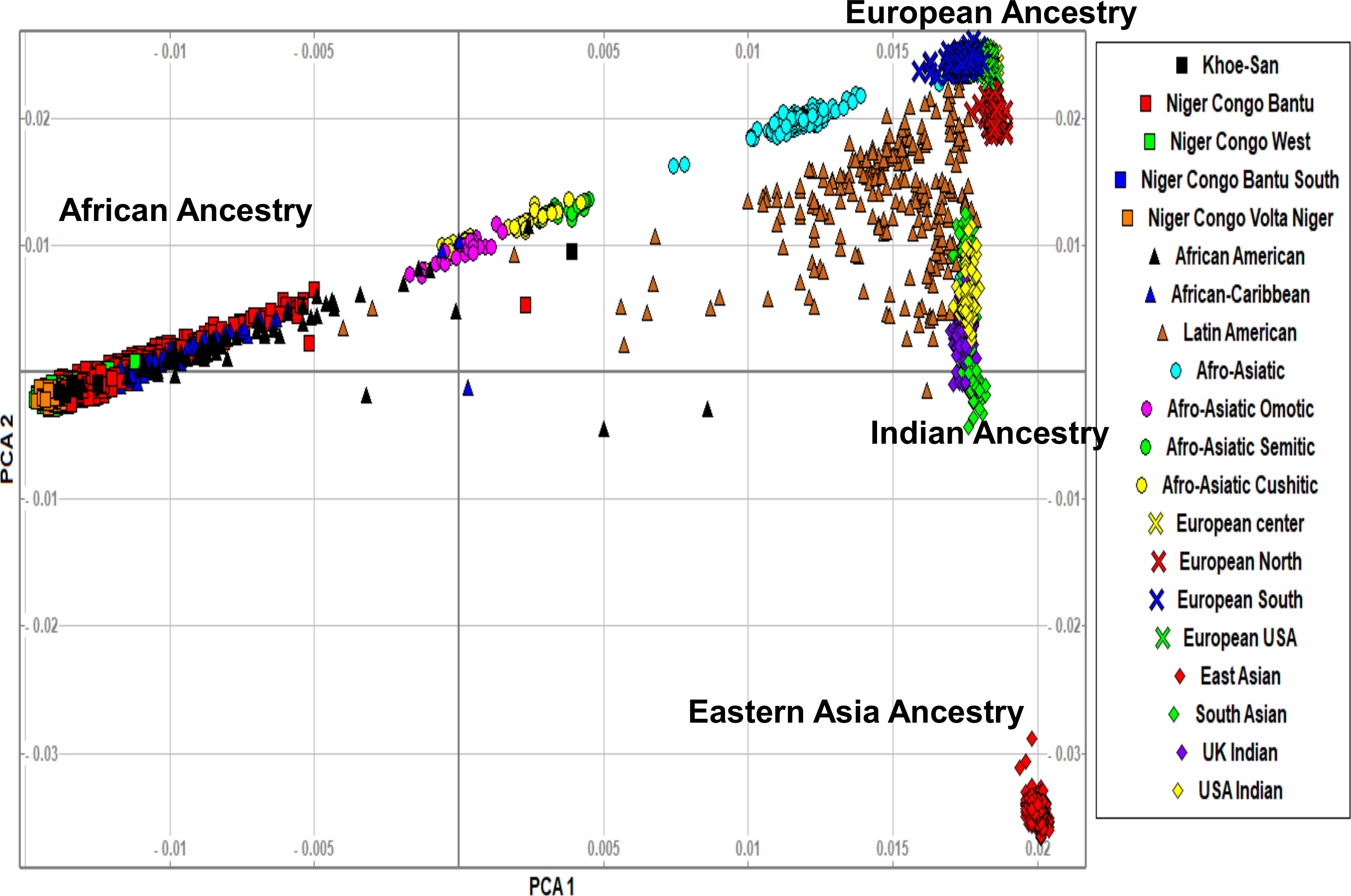

Supplement: Supplementary file 7 [file Image1.TIF]
